# Cardiolipin Synthesis in Skeletal Muscle Is Rhythmic and Modifiable by Age and Diet

**DOI:** 10.1155/2020/5304768

**Published:** 2020-06-14

**Authors:** Kazunari Nohara, Eunju Kim, Marvin Wirianto, Eugenia Mileykovskaya, William Dowhan, Zheng Chen, Seung-Hee Yoo

**Affiliations:** Department of Biochemistry and Molecular Biology, The University of Texas Health Science Center at Houston, 6431 Fannin St., Houston, TX 77030, USA

## Abstract

Circadian clocks regulate metabolic processes in a tissue-specific manner, which deteriorates during aging. Skeletal muscle is the largest metabolic organ in our body, and our previous studies highlight a key role of circadian regulation of skeletal muscle mitochondria in healthy aging. However, a possible circadian regulation of cardiolipin (CL), the signature lipid class in the mitochondrial inner membrane, remains largely unclear. Here, we show that CL levels oscillate during the diurnal cycle in C2C12 myotubes. Disruption of the *Ror* genes, encoding the ROR nuclear receptors in the secondary loop of the circadian oscillator, in C2C12 cells was found to dampen core circadian gene expression. Importantly, several genes involved in CL synthesis, including *Taz* and *Ptpmt1*, displayed rhythmic expression which was disrupted or diminished in Ror-deficient C2C12 cells. *In vivo* studies using skeletal muscle tissues collected from young and aged mice showed diverse effects of the clock and aging on the oscillatory expression of CL genes, and CL levels in skeletal muscle were enhanced in aged mice relative to young mice. Finally, consistent with a regulatory role of RORs, Nobiletin, a natural agonist of RORs, was found to partially restore transcripts levels of CL synthesis genes in aged muscle under a dietary challenge condition. Together, these observations highlight a rhythmic CL synthesis in skeletal muscle that is dependent on RORs and modifiable by age and diet.

## 1. Introduction

In response to the environmental changes imposed by the daily Earth rotation, diverse organisms are known to display intrinsic circadian (approximately daily) rhythms, including sleep/wake cycles, feeding/fasting alternation, and rhythmic fluctuation in various metabolic and physiological processes such as heart rate, body temperature, hormone secretion, locomotion, and cognition [1]. The timing device driving these circadian rhythms is the circadian clock that has evolved in various organisms [2]. In mammals, the clock is organized in a hierarchical manner, with cellular oscillators synchronized by the brain master pacemaker in the hypothalamic suprachiasmatic nuclei (SCN) driving gene expression and output physiology throughout the body [3]. As we age, there is a marked decline in circadian robustness, as evidenced by altered central-peripheral communication, dampened circadian transcriptome, and systemic age-related deterioration in various clock-controlled processes [4]. Understanding the molecular target and regulatory pathways underlying the clock and clock outputs in the context of aging is a critical question.

The basic functional unit of the mammalian circadian clock is the cellular oscillators comprised of interconnected feedback loops [5]. In the core loop, CLOCK/BMAL1 and NPAS2/BMAL1 heterodimeric transcription factor complexes drive the transcription of *Period1/2/3* and *Cryptochrome1/2* genes (denoted as *Per1/2/3* and *Cry1/2*, respectively). The protein products, PERs and CRYs, subsequently heterodimerize and translocate to the nucleus to inhibit the BMAL1-containing complexes and thus shutting down their own transcription. This core loop intersects with several other additional loops to perform highly precise timekeeping with a ~24 hr periodicity. A major additional loop is the stabilization loop (also known as secondary loop), where competing nuclear receptor families (REV-ERBs and RORs) antagonize each other in binding to the RORE elements of several clock genes, including *Bmal1* and *Npas2*, to inhibit or activate their transcription, respectively [5–8]. Expression of *Nr1d1/2* (also called *Rev-erba/b*, encoding REV-ERB*α*/*β*) and *Rora/b/c* (encoding ROR*α*/*β*/*γ*) is also regulated by BMAL1, completing the feedback loop. The secondary loop is believed to confer stability and robustness for the oscillator, playing an important role in circadian amplitude regulation. In our previous studies [9–11], we showed that the natural compound Nobiletin (NOB), acting as an agonist of the positive factor RORs in the secondary loop, was able to enhance core oscillator amplitude and confer protection against various metabolic and dietary challenges in both young and old mice.

It is well established that the clock and metabolism are closely connected [12, 13]. On the one hand, metabolism regulates circadian clocks in many ways. For example, high-caloric intake is known to dampen the circadian clock amplitude [14]. On the other hand, a fundamental, evolutionarily conserved function of the circadian clock is metabolic regulation. In nonphotosynthetic organisms such as mammals, the clock still plays a central role to orchestrate metabolic functions. Transcriptomic analysis, including pioneering studies of liver and the SCN and the more recent comprehensive profiling in mice and baboon, has illustrated prevalent yet highly tissue-specific circadian transcriptome [15–17]. While only ~10% genes in specific tissues display circadian expression, they tend to be regulatory nodal points such as master transcription factors/cofactors, rate-limiting enzymes, and nutrient sensors, indicating an effective strategy of the clock to control metabolic flux over the daily cycle. Of note, skeletal muscle, as the largest metabolic organ in our body, is increasingly appreciated as a key circadian clock target [18, 19]. For example, skeletal muscle is the major site of glucose disposal; when the muscle clock is disrupted via *Bma1* knockout (KO), several steps in the fuel entry and utilization steps were found to be dysregulated, leading to impaired glucose homeostasis [20]. Further, global *ClockΔ19* mutation and *Bmal1* KO led to impaired muscle structure and physiology, and interestingly, muscle mitochondrial volume and function were found to be strongly attenuated in these mutant mice [21].

As the powerhouse of energy metabolism, mitochondria are tightly controlled by the clock in various ways [22, 23]. As mentioned above, fuel entry to muscle mitochondria is controlled at the pyruvate dehydrogenase level [20]. In mouse liver, the rate of fatty acid oxidation (FAO) in the mitochondria showed diurnal patterns, and it was found that SIRT3, the sirtuin subtype with a prominent role in the mitochondria, was controlled by the clock [24]. Lipidomics analysis revealed that several lipid classes showed circadian oscillation in the mitochondria [25]. The dimeric phospholipid Cardiolipin (CL) is specifically localized to the mitochondrial inner membrane in the eukaryotes [26, 27]. Characterized by a small head group and four bulky polyunsaturated acyl chains, CL is known to be critical for mitochondrial membrane structure, cristae formation, membrane protein binding, and structural integrity of mitochondrial respiratory chain complexes and supercomplexes [28]. CL undergoes a multistep synthetic pathway that is highly conserved from yeasts to mammals, where a series of enzymes are involved in forming the backbone, attaching the acyl chains, and remodeling the chains to generate the final products [26]. In previous yeast genetic and biochemical studies, it was demonstrated that manipulation of CL pathway gene expression, and consequently CL levels and composition, has strong effects on mitochondrial respiratory supercomplex formation [29–34]. Furthermore, mammalian studies showed that altered levels of CL or acyl lipid composition affect mitochondrial respiration in various pathological conditions, including the Barth Syndrome, metabolic and cardiovascular disorders, and aging [26, 28, 35, 36].

Despite mitochondria being a highly clock-regulated organelle with CL being the signature lipid in the mitochondria, very little is known about a potential regulatory role of the clock in CL homeostasis. Here, using both *in vitro* (C2C12 myotubes) and *in vivo* (young and aged mice) systems, we provide evidence supporting an important role of the clock, particularly RORs, in CL synthesis, which can be modified by age and diet.

## 2. Materials and Methods

### 2.1. C2C12 Cell Culture, Differentiation and CRISPR

C2C12 cells (ATCC) were maintained in DMEM with 10% FBS and penicillin/streptomycin until 80-90% confluence. For differentiation, cells were incubated in differentiation media (DM; DMEM containing 2% horse serum and penicillin/streptomycin). DM was changed daily until cells were fully differentiated (around day 5). To generate Rorac CRISPR deletion cell lines, the sense and antisense gRNAs were designed using the https://crispr.dbcls.jp/ program (Table 1) and cloned into the BsmB1 site of the GeCKO vector [37] for C2C12 transfection followed by puromycin selection. To examine clock gene expression, C2C12 cells were differentiated in DM until day 2. Cells were treated with 100 nM of Dexamethasone (Dex) for 1 hr for synchronization on day 2. After release from Dex treatment (CT0), cells were harvested and lysed in TRIzol reagent every 4 hrs. Samples were stored in -80°C before qPCR analysis.

### 2.2. Cardiolipin Assays

**Figure 1:**
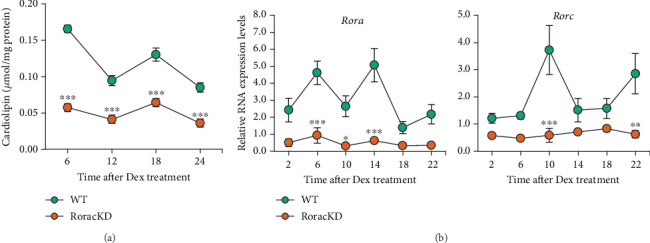
Cardiolipin levels in C2C12 myotube cells over a circadian cycle. (a) CL levels in wild-type (WT) control and *Ror* double knockdown (KD) C2C12 cells were measured by a fluorometric assay and showed a strong reduction in level and oscillation compared with WT (*n* = 3). (b) CRISPR knockdown of *Ror* in C2C12 cells. Representative real-time qPCR results showing knockdown of *Rora* (left) and *Rorc* (right) in C2C12 cells over a circadian cycle (*n* = 3). Data are presented as mean ± SEM. ∗*p* < 0.05, ∗∗*p* < 0.01, ∗∗∗*p* < 0.001, two-way ANOVA with Sidak multiple comparisons test. Circadian time (CT) 0 indicates the time of Dexamethasone treatment.

**Figure 2:**
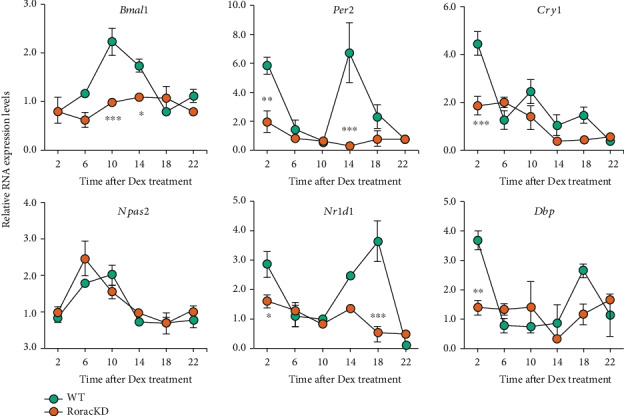
Ror deletion alters circadian gene cycling in C2C12 cells. Real-time qPCR analysis of circadian gene expression in WT and *Rorac* double knockdown (KD) C2C12 cells over a circadian cycle (*n* = 3). Data are presented as mean ± SEM. ∗*p* < 0.05, ∗∗*p* < 0.01, ∗∗∗*p* < 0.001, two-way ANOVA with Sidak multiple comparisons test, showing significant statistical differences between WT and Rora/c double knockdown (KD) for *Bmal1*, *Per2*, *Cry1*, *Nr1d1* (or *Rev-erba*), *Dbp*, and *Npas2*.

**Figure 3:**
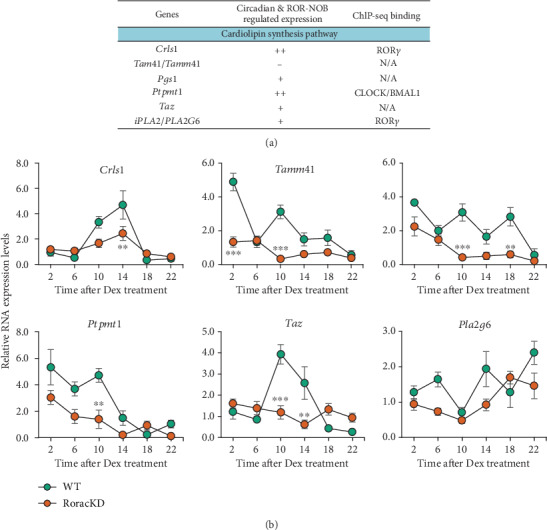
Identification of potential CL pathway genes regulated by ROR and the clock. (a) Genes encoding components required for CL synthesis were examined for oscillatory expression and promoter binding by circadian proteins were examined based on cross-comparison between published database and our own gene expression studies. In the middle column, we assign the symbols to reflect the possibility of circadian regulation of its expression based on existing data. N/A: data not available. (b) Real-time qPCR analysis of CL pathway genes in WT and RoracKD C2C12 cells over a circadian cycle (*n* = 3). Data are presented as mean ± SEM. ∗*p* < 0.05, ∗∗*p* < 0.01, ∗∗∗*p* < 0.001, two-way ANOVA with Bonferroni multiple comparisons test.

Cardiolipin contents in skeletal muscle or C2C12 culture cells were measured by fluorometric assay (BioVision; Catalog # K944-100). In brief, tissue samples or cultured cells were sonicated in a proprietary extraction buffer (Figures 1 and 2) or PBS (Figure 3). After centrifugation for 10 min at 10,000 g and 4°C, supernatants were transferred to new centrifuge tubes on ice. Protein concentrations were measured by using a Coomassie protein assay kit (Thermo Scientific; #1856209). Cardiolipin assays were performed following the manufacturer's protocol. The fluorescence was measured at an excitation wavelength of 340 nm and an emission wavelength of 480 nm.

### 2.3. Circadian ChIP-Seq Database Search

We examined RORs/REV-EEB*α* or CLOCK/BMAL1 promoter binding by cross-checking ChIP-seq databases [38–40] with our circadian and NOB-regulated RNA-seq results [10].

### 2.4. Real-Time qPCR

RT-qPCR analysis was conducted as previously described with minor modifications [41]. Total RNA was extracted from frozen calf muscle by applying the TRizol method (Invitrogen). One or 2 *μ*g of extracted RNA were used for cDNA synthesis. Gene expression was analyzed by using Mx3000p (Agilent technologies). Primer sequences are listed in Table 2.

### 2.5. Animal Studies

Animal studies were conducted as previously described [10, 11]. Briefly, young (6 weeks) and aged (20-22 months) male C57BL/6 mice were obtained from the Jackson Laboratory (#000664) and the aged mouse colony at the National Institute on Aging (NIA), respectively. Mice were maintained under normal 12 hr : 12 hr light : dark cycles in central institutional facilities and fed a regular diet (RD, Purina 5053 from Pico Lab) unless otherwise indicated. Zeitgeber time (ZT) 0 refers to light on (7 am). After 2-3 weeks of acclimation, animals were treated as follows. In the HF feeding experiments, HF and HF.NOB were purchased from Research Diets (D12492 and D14081401, 0.1% NOB). Both RD and HF groups were maintained for 20-24 weeks before tissue collection under the normal light : dark cycles. NOB was obtained from commercial sources (GenDEPOT and Selleck Chem.). Calf muscle used throughout this study refers to mixed type gastrocnemius and soleus muscles. All animal studies were approved by UTHealth Center for Laboratory Animal Medicine and Care (CLAMC).

### 2.6. Quantification and Statistical Analysis

Results are presented as mean ± SEM unless otherwise stated. Data were analyzed using student's *t*-test, one-way ANOVA followed by post hoc analysis using *Dunnett's* multiple comparisons test or two-way ANOVA followed by post hoc analysis using Sidak or Bonferroni test as appropriate.

## 3. Results

### 3.1. ROR-Dependent Diurnal Rhythm of CL Levels in C2C12 Myotubes

To investigate a possible CL oscillation in mouse skeletal muscle, we first employed an *in vitro* system, i.e., C2C12 cells. Using differentiation media containing 2% horse serum, we differentiated C2C12 into myotubes. The cells were subsequently synchronized by Dexamethasone, and CL levels were measured by fluorometric assays. As shown in Figure 1(a), CL levels displayed a bimodal rhythm in wild-type C2C12 myotubes (green trace).

RORs are key regulators in the secondary loop for circadian oscillatory robustness. To investigate a potential role of RORs in the rhythmic pattern of CL, we performed CRISPR to delete *Rora* and *Rorc* (Figure 1(b)). The *Ror*-disrupted cells (RoracKD, red trace) displayed markedly lower CL levels at all CTs compared to WT, as well as significant dampening of the peak CL levels and overall amplitude (Figure 1(a)). These CL effects are in accordance with the recently reported mitochondrial impairments in these cells [10]. Together, these results reveal a diurnal rhythm of CL in myotubes, and the oscillation is modulated by RORs.

### 3.2. Core Clock Gene Expression in C2C12 Cells

To further investigate how CL oscillation is regulated by the circadian machinery, we first characterized the expression of clock genes in the core oscillator. As expected, we observed robust circadian oscillations of core clock gene expression in C2C12 myotubes with characteristic phase relationships (Figure 2). For example, *Bmal1* and *Npas2*, both ROR target genes, showed peak expression at CT10, whereas transcript levels of *Per2*, a target of *Bmal1* and *Npas2*, showed a distinct pattern with the nadir at CT10 in the WT cells. Next, we examined the effects of Ror deletion on clock genes in RoracKD cells. Interestingly, we observed marked reductions in the oscillatory amplitude as a result of *Rora/c* ablation. Surprisingly, the amplitude of *Npas2* expression was not altered, yet the phase was advanced. These results show a critical role of RORs in maintaining normal amplitude and phase of core clock gene expression.

### 3.3. ROR Requirement for CL Gene Expression

We next examined the regulatory role of RORs for CL synthesis genes. Previously, ChIP-seq analyses have been performed for circadian factors, including BMAL1 (E-box binding) and REV-ERB and ROR (RORE binding) [38, 39, 42–44]. We therefore first conducted database search to examine circadian factor binding to the promoters of CL synthesis genes. As shown in Figure 3(a), several CL synthesis genes have been found to display clock protein binding on their promoters. Specifically, *Ptpmt1* showed CLOCK/BMAL1 binding, and ROR*γ* was found to bind to *Crls1* and *iPla2* promoters.

We therefore examined these and other genes involved in CL synthesis in C2C12 myotubes. A reduction in gene expression was observed in several genes (Figure 3(b)), consistent with a positive transactivation function of RORs. However, the degree of alteration did not strictly correlate with the promoter binding as revealed by ChIP-seq analysis. For example, although *Taz*, a gene required for CL remodeling, was not previously found to display either BMAL1 or ROR*γ* binding, *Ror* ablation resulted in both strong reductions in expression levels and marked dampening of circadian oscillation (Figure 3(b)). Together, these results suggest a complex role of the circadian clock and RORs in the regulation of CL genes in C2C12 cells.

### 3.4. CL Level and CL Synthesis Gene Expression in Young and Aged Skeletal Muscle

**Figure 4:**
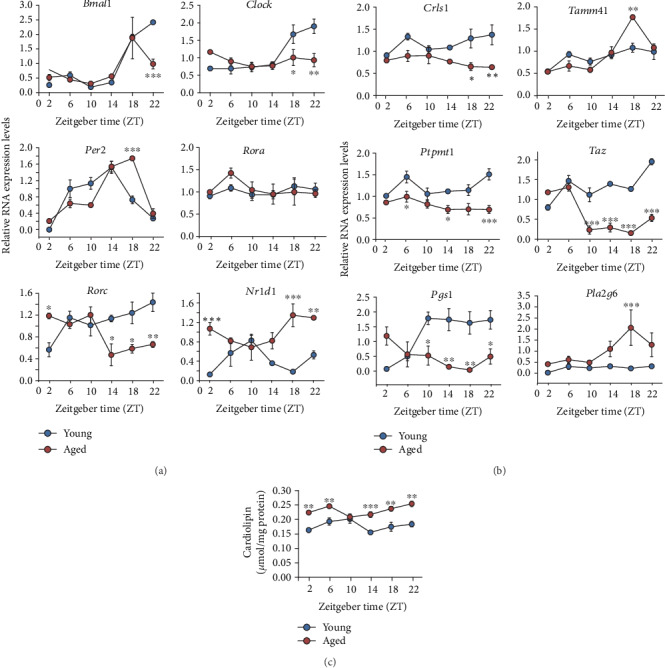
CL pathway gene expression and CL levels in young and aged mouse skeletal muscle. Calf muscle tissues were collected over a circadian cycle from young (2-3 months) and aged (20-22 months) mice. (a) qPCR analysis of core clock gene expression over the circadian cycle (*n* = 3). (b) qPCR analysis of CL pathway gene expression over the circadian cycle (*n* = 3). (c) CL levels over the circadian cycle were measured by fluorometric assays (*n* = 3). Data are presented as mean ± SEM. ∗*p* < 0.05, ∗∗∗*p* < 0.001, one-way ANOVA with Dunnett's multiple comparisons test or ^##^*p* < 0.01, student's *t*-test.

Next, we examined CL synthesis *in vivo*. To examine aging effects, we subjected both young (2-3 months) and aged (20-22 months) old mice to normal husbandry, including regular diets and 12 : 12 light : dark cycles. Calf muscle tissues were collected over a full circadian time course for gene expression and cardiolipin assays. We first performed qPCR analysis to examine circadian and CL gene expression. As shown in Figure 4(a), the overall expression of two core loop genes, *Bmal1* and *Per2*, was not significantly altered by aging. On the other hand, *Rorc* and *Nr1d1*, two genes in the secondary loop, showed more marked alteration between young and old mice. Specifically, their expression patterns were antiphasic and differentially affected by aging, with *Rorc* expression reduced and *Nr1d1* elevated in aged mice vs young mice. Such opposing expression patterns are consistent with their antagonistic transcriptional functions on RORE promoters (Figure 4(a)).

Compared with the diverse age-dependent effects in core clock genes, several CL synthesis genes showed considerable reduction in aged muscle, including *Crls1*, *Ptpmt1,* and *Taz* (Figure 4(b)). However, the peak expression of *Tamm41* was strongly enhanced at CT15, suggesting a complex circadian and aging effect. We then measured CL levels in young and aged muscle by fluorometric assays (Figure 4(c)). CL levels showed a bimodal oscillation in the calf muscle of young mice, and somewhat surprisingly were generally higher and displayed a distinct phase pattern in aged mice relative to young mice. In conjunction with previous studies [25, 45, 46], this result further suggests that aging and the circadian clock exert a complex role in CL regulation (see Discussion).

### 3.5. An ROR Agonist, Nobiletin (NOB), Activates CL Gene Expression in Metabolically Challenged Aged Mice

We previously identified via high-throughput chemical screening a naturally occurring compound called Nobiletin (NOB) as an ROR agonist [9]. NOB was found to display robust metabolic efficacies in high-fat (HF) diet-fed obese young and aged mice, in part via NOB induction of ROR target genes [9–11, 47]. Particularly, in aged mice challenged with HF feeding, NOB was found to markedly improve mitochondrial function to approximate the level observed in unchallenged aged mice (RD-fed) [10]. We therefore examined gene expression at Zeitgeber time (ZT) 6 and 18, corresponding to daytime and nighttime, in aged mice fed with HF diet with or without NOB, with RD feeding as a control as previously described [10].

**Figure 5:**
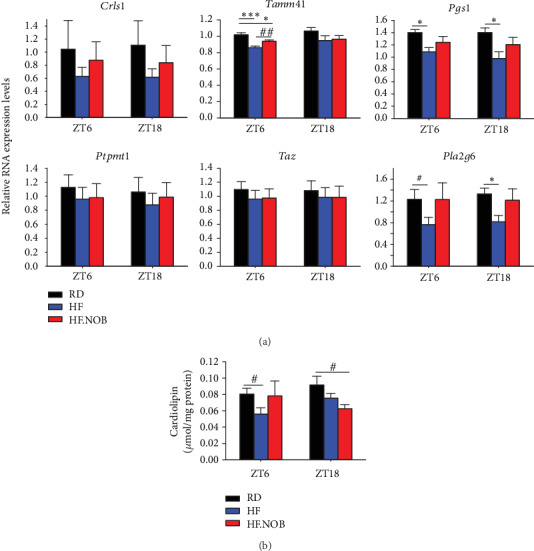
NOB treatment showed beneficial effects to partially restore CL gene expression and CL levels under the high-fat (HF) condition. Aged mice were fed with regular diet (as a control) or HF diet with or without NOB. (a) Effects of HF diet and NOB on the expression of CL synthesis genes (*n* = 7 − 9). Data are presented as mean ± SEM. ∗*p* < 0.05, ∗∗*p* < 0.01, ∗∗∗*p* < 0.001, one-way ANOVA with Dunnett's multiple comparisons test. (b) Effects of HF diet and NOB on CL levels (*n* = 4 − 6). Note that the sample extraction was done by using PBS. Data are presented as mean ± SEM. ^#^*p* < 0.05, student's *t*-test.

Compared to the RD condition, HF feeding attenuated expression of several CL pathway genes in aged mice, including *Tamm41* and *Pgs1* (Figure 5(a)). Interestingly, NOB treatment in the presence of HF was able to recover, significantly or with a trend, their expression to approach levels under the RD control condition, especially at ZT6. In accordance, CL levels under the HF condition were found to be reduced relative to the control (Figure 5(b)), whereas NOB treatment showed a trend to recover it at ZT6. This rescue effect is consistent with the overall mitochondria-enhancing function of NOB in metabolically challenged aged mice [10].

Together, these results demonstrate significant effects of HF diet and NOB on CL gene expression under the aging condition, consistent with a key regulatory role of RORs in CL synthesis.

## 4. Discussion

In the current study, we show that CL levels and expression of several CL synthesis genes display oscillatory patterns in C2C12 myotubes in an ROR-dependent manner. Ror-disrupted C2C12 cells showed diminished amounts and altered rhythms of CL, consistent with the previously observed mitochondrial dysfunction, including reduced ATP production and mitochondria content, in these cells [10]. *In vivo* studies revealed diverse effects of the clock and aging on CL gene expression and CL levels in skeletal muscle. Furthermore, we observed a rescuing effect of the ROR agonist Nobiletin (NOB) on CL gene expression and CL levels in skeletal muscle from HF-fed aged mice. Together, these results indicate that the clock and RORs contribute to muscle CL synthesis in a process modifiable by age and diet, underscoring a novel circadian controlled process in mitochondrial function.

Despite active research showing robust circadian regulation of various aspects of mitochondrial dynamics and function, CL, as the signature lipid of mitochondria, has not been shown to be directly regulated by the clock. In pioneering studies [48], CL levels appeared to display diurnal rhythms in rat brain. More recent lipidomics studies of liver and skeletal muscle mitochondria showed moderate oscillatory patterns for CL levels [25, 49]. In mouse heart, a recent study showed that levels of CL76:11 and CL72:8 differentially accumulate in an unknown mechanism regulated by circadian disruption and sex [50]. To directly investigate a circadian regulation of CL as opposed to diurnal rhythms, the current work focused on CL in skeletal muscle and observed clear oscillations both *in vitro* and *in vivo*. Importantly, our study elucidates circadian oscillation of several genes involved in CL synthesis and demonstrates a regulatory role of the ROR nuclear receptors, essential components of the secondary loop in the circadian cellular oscillator, in the circadian expression of these genes. Together, our results unveil circadian accumulation of CL in a mitochondria-rich metabolic organ and provide a molecular basis underlying CL dynamics.

Mitochondrial dysregulation is a well-recognized hallmark of aging [51]. In addition to mitochondrial dynamics and respiration, one emerging aspect of mitochondrial aging is dysregulation of CL. For example, it has been proposed that oxidative stress and CL peroxidation contributes to reduced levels of individual respiratory chain complexes and supercomplexes [52]. As a fundamental guardian of well-being, circadian timing is increasingly appreciated to reciprocally interact with the aging process [4]. On the one hand, with age, the clock machinery is less synchronized, and the output gene expression and physiology are less robust [53–55]. On the other hand, the clock state can be manipulated to influence aging [56–59]. Genetic and environmental disruption of the clock has been shown to accelerate aging. For example, while imposing a jet-lag paradigm in mice greatly compromised health and exacerbated mortality [60, 61], several dietary interventions, including CR and TRF, have been shown to alter circadian timing and promote healthy aging [57, 62, 63]. In many of these circadian aging studies, mitochondrial functional modulation has been observed. Whereas mitochondrial function was impaired in *Bmal1* KO mice in correlation with a premature aging phenotype [59], mitochondrial respiration chain genes were downregulated in TRF-treated flies with improved cardiac function at old ages [63], suggesting a complex relationship between mitochondrial functional state and aging, and a modulatory role of the clock in this process.

RORs represent the positive arm of the secondary loop, competing with REV-ERBs to activate expression of both core clock genes (e.g., *Bmal1* and *Npas2*) and clock-controlled genes. Previously, RORs have been shown to play important roles in tissue and systemic metabolism [42, 64, 65]. For example, a pioneering study previously showed that genes involved in lipid homeostasis, including *Cpt-1* and *caveolin-3*, are directly controlled by RORs in mouse skeletal muscle [65]. Various endogenous and exogenous ligands have been found to bind to RORs, particularly the ligand-binding domain, and exert a broad range of physiological functions including metabolism and immunity [66–71]. Previous studies have identified a natural compound, Nobiletin (NOB), as a potent clock modulator, specifically acting as an ROR agonist to activate various ROR target genes in accordance with a beneficial role of NOB in preventing metabolic disease [9, 47, 72, 73]. In the current study, we further showed that CL synthesis is a new cellular pathway target for RORs, as evidenced by altered or dampened expression in the absence of RORs. NOB treatment of aged mice was found to enhance CL gene expression *in vivo*, providing a physiological context where CL regulation by RORs and the clock can promote health and healthspan. This is of particular interest because NOB has previously been shown to serve as an antioxidant agent [10, 74, 75], although the cellular mechanisms were not well understood. Our study raises the intriguing possibility that NOB may function to suppress oxidative stress via CL regulation. Future studies will investigate the detailed molecular and transcriptional mechanisms of how the ROR-NOB axis regulates CL gene expression.

Several key questions require further investigations. It is somewhat surprising to observe elevated CL levels in aged muscle tissues compared to young ones, whereas CL gene expression was found to be attenuated in aged tissues. While it is well recognized that gene expression at the mRNA level may be uncoupled from protein level/activity and consequently the downstream process (CL level in this case), there are other potentially important contributing factors. First, age-related changes in skeletal muscle may be relevant. The CL assays used muscle protein amounts to normalize the relative CL level. Muscle mass decreases over age (sarcopenia); furthermore, type 1 and type 2 muscle fibers are distinct in aging rate and CL content, with the latter aging faster and less abundant in CL [76]. These aging-induced alterations could conceivably affect the normalization and calculation of CL abundance in our assays using the mixed type calf muscle. Second, mitochondrial aging is a complex process where different aspects of mitochondrial composition and function are modulated by numerous factors including diet/exercise, tissue type, and species [45, 46, 51, 77]. In a previous study, aged rat brains were found to have decreased CL levels and increased proportions of peroxidized CL [45]. In addition, aged mouse skeletal muscle showed robust induction of CL species (9.3 and 7.5 fold for CL (72 : 10) and CL (70 : 6), respectively), consistent with the notion that CL levels can increase over age, at least under certain contexts. Such induction may be a compensatory mechanism to maintain mitochondrial function in an energy-demanding organ at old ages. More generally, how the regulation of CL by the clock and RORs may translate into physiological benefits should be further investigated. CL is localized in the energy-transducing mitochondrial inner membrane which maintains proton gradient. With its conical shape and dimeric structure, CL is known to promote interactions with individual proteins and their associations into supramolecular complexes, potentially functioning as glue to fill the cavity at the interface of complex interaction [26, 27, 78]. Therefore, it would be informative in future studies to examine the composition and peroxidation state of CL, as well as mitochondrial morphology and dynamics, in different tissues and ages. Of note, in a recent study [10], our results showed improved mitochondrial content, respiration activity, and selective supercomplex formation in HF-fed aged mice treated with NOB in a circadian time-dependent manner. Overall, the observations reported here are well aligned with the functional data therein, in strong support of an important role of mitochondrial enhancement by the circadian clock to promote healthy aging. Other functional benefits are also possible, including a potential antiapoptotic role of CL [79].

## 5. Conclusion

In conclusion, we herein reveal a clock- and ROR-dependent regulatory mechanism for CL diurnal rhythm in mouse skeletal muscle which can be modified by aging and diet. Future studies will probe the underlying molecular mechanism and determine the physiological function.

## Figures and Tables

**Table 1:** CRISPR primers. All sequences are 5′-3′.

gRNA target	Sequence
Rora-1f	caccgAGAGACAGCTTGTACGCCG
Rora-1r	aaacCGGCGTACAAGCTGTCTCTc
Rora-2f	caccgACCTCAGCACCTATATGGA
Rora-2r	aaacTCCATATAGGTGCTGAGGTc
Rora-3f	caccgAAACCGCTGCCAGCATTGT
Rora-3r	aaacACAATGCTGGCAGCGGTTTc
Rorc-1f	caccgTGATCCCTTGCAAGATCTG
Rorc-1r	aaacCAGATCTTGCAAGGGATCAc
Rorc-2f	caccgCAGCAGCAACAGGAACAAG
Rorc-2r	aaacCTTGTTCCTGTTGCTGCTGc
Rorc-3f	caccgACAGCATCTATAGCACTGA
Rorc-3r	aaacTCAGTGCTATAGATGCTGTc

**Table 2:** Primers used for real-time qPCR. All sequences are 5′-3′.

	**Forward**	**Reverse**
*Crls1*	ATGCCACGGCTAGGTTAAAAC	GCTGTGCAACACCATAGTATCT
*Tamm41*	CTAGTCGCACCACATCTCCA	AAGCCCAGAAGGACAGTTCA
*Ptpmt1*	ACTATGAACGAGGAGTACGAGAC	GTTGGGACCCCAGTCATGTC
*Taz*	CCCCCGCTTTGGACAGAAAAT	AGGCTGGAAATGATTGTGGAG
*Pgs1*	CCCACCTTGCTGCCTATGTC	GCCATCACAACTCGCCTCT
*Pla2g6*	CGGCCTGAACCAGGTAAACAA	GTTGCAGCGGGCATTACAG
*Clock*	CCTTCAGCAGTCAGTCCATAAAC	AGACATCGCTGGCTGTGTTAA
*Bmal1*	CCACCTCAGAGCCATTGATACA	GAGCAGGTTTAGTTCCACTTTGTCT
*Per2*	ATGCTCGCCATCCACAAGA	GCGGAATCGAATGGGAGAAT
*Cry1*	CTGGCGTGGAAGTCATCGT	CTGTCCGCCATTGAGTTCTATG
*Npas2*	CAACAGACGGCAGCATCATCT	TTCTGATCCATGACATCCGC
*Rora*	GCACCTGACCGAAGACGAAA	GAGCGATCCGCTGACATCA
*Rorc*	TCAGCGCCCTGTGTTTTTC	GAGAACCAGGGCCGTGTAG
*Nr1d1*	CATGGTGCTACTGTGTAAGGTGTGT	CACAGGCGTGCACTCCATAG
*Dbp*	CTGGCCCGAGTCTTTTTGC	CCAGGTCCACGTATTCCACG
*Gapdh*	CAAGGTCATCCATGACAACTTTG	GGCCATCCACAGTCTTCTGG
*Actb*	TTGTCCCCCCAACTTGATGT	CCTGGCTGCCTCAACACCT

## Data Availability

Original data are available upon request.

## References

[1] Bass J. (2012). Circadian topology of metabolism. *Nature*.

[2] Bell-Pedersen D., Cassone V. M., Earnest D. J. (2005). Circadian rhythms from multiple oscillators: lessons from diverse organisms. *Nature Reviews. Genetics*.

[3] Mohawk J. A., Green C. B., Takahashi J. S. (2012). Central and peripheral circadian clocks in mammals. *Annual Review of Neuroscience*.

[4] Manoogian E. N. C., Panda S. (2017). Circadian rhythms, time-restricted feeding, and healthy aging. *Ageing Research Reviews*.

[5] Takahashi J. S. (2017). Transcriptional architecture of the mammalian circadian clock. *Nature Reviews. Genetics*.

[6] Dibner C., Schibler U., Albrecht U. (2010). The mammalian circadian timing system: organization and coordination of central and peripheral clocks. *Annual Review of Physiology*.

[7] Preitner N., Damiola F., Luis-Lopez-Molina (2002). The Orphan Nuclear Receptor REV-ERB*α* Controls Circadian Transcription within the Positive Limb of the Mammalian Circadian Oscillator. *Cell*.

[8] Sato T. K., Panda S., Miraglia L. J. (2004). A functional genomics strategy reveals Rora as a component of the mammalian circadian clock. *Neuron*.

[9] He B., Nohara K., Park N. (2016). The small molecule nobiletin targets the molecular oscillator to enhance circadian rhythms and protect against metabolic syndrome. *Cell Metabolism*.

[10] Nohara K., Mallampalli V., Nemkov T. (2019). Nobiletin fortifies mitochondrial respiration in skeletal muscle to promote healthy aging against metabolic challenge. *Nature Communications*.

[11] Nohara K., Nemkov T., D’Alessandro A., Yoo S. H., Chen Z. (2019). Coordinate regulation of cholesterol and bile acid metabolism by the clock modifier nobiletin in metabolically challenged old mice. *International Journal of Molecular Sciences*.

[12] Green C. B., Takahashi J. S., Bass J. (2008). The meter of metabolism. *Cell*.

[13] Reinke H., Asher G. (2019). Crosstalk between metabolism and circadian clocks. *Nature Reviews. Molecular Cell Biology*.

[14] Kohsaka A., Laposky A. D., Ramsey K. M. (2007). High-fat diet disrupts behavioral and molecular circadian rhythms in mice. *Cell Metabolism*.

[15] Panda S., Antoch M. P., Miller B. H. (2002). Coordinated transcription of key pathways in the mouse by the circadian clock. *Cell*.

[16] Zhang R., Lahens N. F., Ballance H. I., Hughes M. E., Hogenesch J. B. (2014). A circadian gene expression atlas in mammals: implications for biology and medicine. *Proceedings of the National Academy of Sciences of the United States of America*.

[17] Mure L. S., Le H. D., Benegiamo G. (2018). Diurnal transcriptome atlas of a primate across major neural and peripheral tissues. *Science*.

[18] Harfmann B. D., Schroder E. A., Esser K. A. (2015). Circadian rhythms, the molecular clock, and skeletal muscle. *Journal of Biological Rhythms*.

[19] Schroder E. A., Harfmann B. D., Zhang X. (2015). Intrinsic muscle clock is necessary for musculoskeletal health. *The Journal of Physiology*.

[20] Dyar K. A., Ciciliot S., Wright L. E. (2014). Muscle insulin sensitivity and glucose metabolism are controlled by the intrinsic muscle clock. *Molecular Metabolism*.

[21] Andrews J. L., Zhang X., McCarthy J. J. (2010). CLOCK and BMAL1 regulate MyoD and are necessary for maintenance of skeletal muscle phenotype and function. *Proceedings of the National Academy of Sciences of the United States of America*.

[22] de Goede P., Wefers J., Brombacher E. C., Schrauwen P., Kalsbeek A. (2018). Circadian rhythms in mitochondrial respiration. *Journal of Molecular Endocrinology*.

[23] Manella G., Asher G. (2016). The circadian nature of mitochondrial biology. *Frontiers in Endocrinology*.

[24] Peek C. B., Affinati A. H., Ramsey K. M. (2013). Circadian clock NAD+ cycle drives mitochondrial oxidative metabolism in mice. *Science*.

[25] Aviram R., Manella G., Kopelman N. (2016). Lipidomics analyses reveal temporal and spatial lipid organization and uncover daily oscillations in intracellular organelles. *Molecular Cell*.

[26] Mileykovskaya E., Dowhan W. (2014). Cardiolipin-dependent formation of mitochondrial respiratory supercomplexes. *Chemistry and Physics of Lipids*.

[27] Haines T. H. (2009). A new look at Cardiolipin. *Biochimica et Biophysica Acta*.

[28] Pennington E. R., Funai K., Brown D. A., Shaikh S. R. (2019). The role of cardiolipin concentration and acyl chain composition on mitochondrial inner membrane molecular organization and function. *Biochimica et Biophysica Acta - Molecular and Cell Biology of Lipids*.

[29] Mileykovskaya E., Penczek P. A., Fang J., Mallampalli V. K. P. S., Sparagna G. C., Dowhan W. (2012). Arrangement of the respiratory chain complexes in Saccharomyces cerevisiae supercomplex III2IV2 revealed by single particle cryo-electron microscopy. *The Journal of Biological Chemistry*.

[30] Bazán S., Mileykovskaya E., Mallampalli V. K. P. S., Heacock P., Sparagna G. C., Dowhan W. (2013). Cardiolipin-dependent reconstitution of respiratory supercomplexes from purified Saccharomyces cerevisiae complexes III and IV. *The Journal of Biological Chemistry*.

[31] Zhang M., Mileykovskaya E., Dowhan W. (2002). Gluing the respiratory chain together. Cardiolipin is required for supercomplex formation in the inner mitochondrial membrane. *The Journal of Biological Chemistry*.

[32] Zhang M., Mileykovskaya E., Dowhan W. (2005). Cardiolipin is essential for organization of complexes III and IV into a supercomplex in intact yeast mitochondria. *The Journal of Biological Chemistry*.

[33] Pfeiffer K., Gohil V., Stuart R. A. (2003). Cardiolipin stabilizes respiratory chain supercomplexes. *The Journal of Biological Chemistry*.

[34] Azuma K., Ikeda K., Inoue S. (2020). Functional Mechanisms of Mitochondrial Respiratory Chain Supercomplex Assembly Factors and Their Involvement in Muscle Quality. *Int J Mol Sci*.

[35] Pointer C. B., Klegeris A. (2017). Cardiolipin in central nervous system physiology and pathology. *Cellular and Molecular Neurobiology*.

[36] Ghosh S., Iadarola D. M., Ball W. B., Gohil V. M. (2019). Mitochondrial dysfunctions in Barth syndrome. *IUBMB Life*.

[37] Sanjana N. E., Shalem O., Zhang F. (2014). Improved vectors and genome-wide libraries for CRISPR screening. *Nature Methods*.

[38] Koike N., Yoo S. H., Huang H. C. (2012). Transcriptional architecture and chromatin landscape of the core circadian clock in mammals. *Science*.

[39] Takeda Y., Jothi R., Birault V., Jetten A. M. (2012). ROR*γ* directly regulates the circadian expression of clock genes and downstream targets in vivo. *Nucleic Acids Research*.

[40] Feng D., Liu T., Sun Z. (2011). A circadian rhythm orchestrated by histone deacetylase 3 controls hepatic lipid metabolism. *Science*.

[41] Jeong K., He B., Nohara K. (2015). Dual attenuation of proteasomal and autophagic BMAL1 degradation in *Clock*^Δ19/+^ mice contributes to improved glucose homeostasis. *Scientific Reports*.

[42] Zhang Y., Papazyan R., Damle M. (2017). The hepatic circadian clock fine-tunes the lipogenic response to feeding through ROR*α*/*γ*. *Genes & Development*.

[43] Beytebiere J. R., Trott A. J., Greenwell B. J. (2019). Tissue-specific BMAL1 cistromes reveal that rhythmic transcription is associated with rhythmic enhancer-enhancer interactions. *Genes & Development*.

[44] Cho H., Zhao X., Hatori M. (2012). Regulation of circadian behaviour and metabolism by REV-ERB-*α* and REV-ERB-*β*. *Nature*.

[45] Petrosillo G., Matera M., Casanova G., Ruggiero F., Paradies G. (2008). Mitochondrial dysfunction in rat brain with aging: Involvement of complex I, reactive oxygen species and cardiolipin. *Neurochemistry International*.

[46] Pollard A. K., Ortori C. A., Stoger R., Barrett D. A., Chakrabarti L. (2017). Mouse mitochondrial lipid composition is defined by age in brain and muscle. *Aging (Albany NY)*.

[47] Nohara K., Shin Y., Park N. (2015). Ammonia-lowering activities and carbamoyl phosphate synthetase 1 (Cps1) induction mechanism of a natural flavonoid. *Nutrition & Metabolism*.

[48] Díaz-Muñoz M., Suárez J., Hernández-Muñoz R., de Sánchez V. C. (1987). Day-night cycle of lipidic composition in rat cerebral cortex. *Neurochemical Research*.

[49] Loizides-Mangold U., Perrin L., Vandereycken B. (2017). Lipidomics reveals diurnal lipid oscillations in human skeletal muscle persisting in cellular myotubes cultured in vitro. *Proceedings of the National Academy of Sciences of the United States of America*.

[50] Alibhai F. J., Reitz C. J., Peppler W. T. (2018). Female ClockΔ19/Δ19 mice are protected from the development of age-dependent cardiomyopathy. *Cardiovascular Research*.

[51] Lopez-Otin C., Blasco M. A., Partridge L., Serrano M., Kroemer G. (2013). The hallmarks of aging. *Cell*.

[52] Gomez L. A., Hagen T. M. (2012). Age-related decline in mitochondrial bioenergetics: does supercomplex destabilization determine lower oxidative capacity and higher superoxide production?. *Seminars in Cell & Developmental Biology*.

[53] Nakamura T. J., Nakamura W., Yamazaki S. (2011). Age-related decline in circadian output. *The Journal of Neuroscience*.

[54] Sato S., Solanas G., Peixoto F. O. (2017). Circadian reprogramming in the liver identifies metabolic pathways of aging. *Cell*.

[55] Tahara Y., Takatsu Y., Shiraishi T. (2017). Age-related circadian disorganization caused by sympathetic dysfunction in peripheral clock regulation. *npj Aging and Mechanisms of Disease*.

[56] Patel S. A., Chaudhari A., Gupta R., Velingkaar N., Kondratov R. V. (2015). Circadian clocks govern calorie restriction-mediated life span extension through BMAL1- and IGF-1-dependent mechanisms. *The FASEB Journal*.

[57] Acosta-Rodriguez V. A., de Groot M. H. M., Rijo-Ferreira F., Green C. B., Takahashi J. S. (2017). Mice under caloric restriction self-impose a temporal restriction of food intake as revealed by an automated feeder system. *Cell Metab*.

[58] Longo V. D., Panda S. (2016). Fasting, circadian rhythms, and time-restricted feeding in healthy lifespan. *Cell Metabolism*.

[59] Kondratov R. V., Kondratova A. A., Gorbacheva V. Y., Vykhovanets O. V., Antoch M. P. (2006). Early aging and age-related pathologies in mice deficient in BMAL1, the core componentof the circadian clock. *Genes & Development*.

[60] Davidson A. J., Sellix M. T., Daniel J., Yamazaki S., Menaker M., Block G. D. (2006). Chronic jet-lag increases mortality in aged mice. *Current Biology*.

[61] Inokawa H., Umemura Y., Shimba A. (2020). Chronic circadian misalignment accelerates immune senescence and abbreviates lifespan in mice. *Scientific Reports*.

[62] Katewa S. D., Akagi K., Bose N. (2016). Peripheral circadian clocks mediate dietary restriction-dependent changes in lifespan and fat metabolism in Drosophila. *Cell Metabolism*.

[63] Gill S., Le H. D., Melkani G. C., Panda S. (2015). Time-restricted feeding attenuates age-related cardiac decline in Drosophila. *Science*.

[64] Jetten A. M., Kang H. S., Takeda Y. (2013). Retinoic acid-related orphan receptors *α* and *γ*: key regulators of lipid/glucose metabolism, inflammation, and insulin sensitivity. *Frontiers in Endocrinology*.

[65] Lau P., Nixon S. J., Parton R. G., Muscat G. E. O. (2004). ROR*α* regulates the expression of genes involved in lipid homeostasis in skeletal muscle Cells. *The Journal of Biological Chemistry*.

[66] Kojetin D. J., Burris T. P. (2014). REV-ERB and ROR nuclear receptors as drug targets. *Nature Reviews. Drug Discovery*.

[67] Solt L. A., Kumar N., Nuhant P. (2011). Suppression of TH17 differentiation and autoimmunity by a synthetic ROR ligand. *Nature*.

[68] He B., Chen Z. (2016). Molecular targets for small-molecule modulators of circadian clocks. *Current Drug Metabolism*.

[69] Cook D. N., Kang H. S., Jetten A. M. (2015). Retinoic acid-related orphan receptors (RORs): regulatory functions in immunity, development, circadian rhythm, and metabolism. *Nuclear Receptor Research*.

[70] Chang M. R., He Y., Khan T. M. (2015). Antiobesity effect of a small molecule repressor of ROR*γ*. *Molecular Pharmacology*.

[71] Santori F. R., Huang P., van de Pavert S. A. (2015). Identification of natural ROR*γ* ligands that regulate the development of lymphoid cells. *Cell Metabolism*.

[72] Gloston G. F., Yoo S. H., Chen Z. J. (2017). Clock-enhancing small molecules and potential applications in chronic diseases and aging. *Frontiers in Neurology*.

[73] Shinozaki A., Misawa K., Ikeda Y. (2017). Potent Effects of Flavonoid Nobiletin on Amplitude, Period, and Phase of the Circadian Clock Rhythm in PER2::LUCIFERASE Mouse Embryonic Fibroblasts. *PLoS One*.

[74] Umeno A., Horie M., Murotomi K., Nakajima Y., Yoshida Y. (2016). Antioxidative and Antidiabetic Effects of Natural Polyphenols and Isoflavones. *Molecules*.

[75] Mulvihill E. E., Burke A. C., Huff M. W. (2016). Citrus flavonoids as regulators of lipoprotein metabolism and atherosclerosis. *Annual Review of Nutrition*.

[76] Stefanyk L. E., Coverdale N., Roy B. D., Peters S. J., LeBlanc P. J. (2010). Skeletal muscle type comparison of subsarcolemmal mitochondrial membrane phospholipid fatty acid composition in rat. *The Journal of Membrane Biology*.

[77] Mercken E. M., Capri M., Carboneau B. A. (2017). Conserved and species-specific molecular denominators in mammalian skeletal muscle aging. *npj Aging and Mechanisms of Disease*.

[78] Milenkovic D., Blaza J. N., Larsson N. G., Hirst J. (2017). The enigma of the respiratory chain supercomplex. *Cell Metabolism*.

[79] Mulkidjanian A. Y., Shalaeva D. N., Lyamzaev K. G., Chernyak B. V. (2018). Does oxidation of mitochondrial cardiolipin trigger a chain of antiapoptotic reactions?. *Biochemistry (Mosc)*.

